# Editorial: Neurovascular changes in neuroinflammatory diseases

**DOI:** 10.3389/fncel.2022.1075719

**Published:** 2022-11-01

**Authors:** Weidong Cao, Christopher J. Pirozzi, Jian Song, Zenghui Teng

**Affiliations:** ^1^Department of Neurosurgery, The First Medical Centre, Chinese PLA General Hospital, Beijing, China; ^2^The Preston Robert Tisch Brain Tumor Center, Duke University Medical Center, Durham, NC, United States; ^3^Institute of Physiological Chemistry and Pathobiochemistry, Cells-in-Motion Cluster of Excellence, University of Münster, Münster, Germany; ^4^Medical Faculty, Institute of Neuro- and Sensory Physiology, Heinrich Heine University Düsseldorf, Düsseldorf, Germany

**Keywords:** neuroinflammation, blood-brain barrier, cerebrovascular dysfunction, neurodegeneration, glial cells

Immune inflammatory responses have recently been linked to the pathology of disorders of the brain system as epilepsy, Parkinson's disease (PD), and Alzheimer's disease (AD), neuroinflammation impairs the activities of the nervous system and plays a role in the pathogenesis of these diseases (Michael et al., 2014). More problematically, patients with neuroinflammation often have large vascular lesions in the brain, and cerebrovascular dysfunction brought on by the immune inflammatory response can exacerbate the neurological damage.

The purpose of the current issue is to explore how neurovascular changes affect the etiology of neuroinflammatory diseases, which will aid in the identification of novel treatment targets for the aforementioned neurological disorders. Various therapeutic targets would contribute to controlling the development of these diseases or delaying their onset, as well as to repairing or safeguarding the central nervous system.

Additionally, vital elements of the neurovascular unit (NVU) include active microglia and the blood-brain barrier (BBB). The structural and functional integrity of NVU, such as BBB dysfunction or microglia activation, may have an impact on the neuroinflammatory process. One of the causes of neurodegenerative disorders is changes in BBB permeability, which lead to inflammatory responses. Increasing BBB permeability or impairing NVU will cause immune cell migration or cytokine transfer. Peripheral T lymphocyte migration results in the production of pro-inflammatory mediators and the loss of tight junction molecules, including as occludins and adhesion proteins, which may have an impact on the BBB's structural integrity and functionality (Tiedt et al., 2022). Therefore, neurovascular unit regulation is required to CNS neuroinflammation, particularly for above neurodegenerative disorders BBB integrity impairment in Alzheimer's disease accelerate amyloid-beta (Aβ) formation and deposition while slowing Aβ clearance (Bartels et al., 2020).

The neuroinflammation in the brain was connected to the alteration of cognition. In the current issue, according to research by Hu et al., inhibition of brain-derived exosomes (BDEs) release may lessen the cognitive impairment brought on by repetitive mild traumatic brain injury (rmTBI) in mice and offer a new method for enhancing the prognoses for chronic traumatic encephalopathy (CTE), which is influenced by microglial proliferation and activation. Heyburn et al. identified that repeated low-level blast overpressure (BOP) exposure may exacerbate several pathophysiological reactions to brain damage by abruptly altering brain cytokine levels or neurovascular proteins of the blood-brain barrier, thereby affecting behavioral deficits and cognition impairment.

In the central nervous system, neuroinflammation brought on by glial cell activation may aid in the pathological progression of neurodegenerative disorders, which cause cognitive impairment and neuronal dysfunction. By using the bioinformatics method, Pan et al. established a representative sample of the optic nerve head (ONH) at various times after optic nerve crush to analyze the transcriptome data of the optic nerve head (ONH) after optic nerve crush in various glial cells according to their subtype markers (ONC). The genetic codes and modules of these processes hold the key to future research and offer hints for controlling glial cells. Takata et al. draw the conclusion that biological alterations in brain microvascular endothelial cells (BMECs) influence the blood-brain barrier's function and might be associated with the inflammatory response in neurodegenerative disorders. Therefore, a potential treatment for neurodegenerative disease with neuroinflammation would be to maintain or restore BBB integrity by activating signaling pathways in BMECs. Glial cells may play a significant part in the inflammatory process of BBB failure by inducing or controlling the production of cytokines or chemokines.

Alzheimer's disease frequently exhibits neuroinflammation, which aids in the pathogenesis of neurodegenerative illness of the brain. Tau and Aβ pathology in AD are caused by neuroinflammation brought on by activated microglia. Through the activation of microglia in the brain, the following cytokines or chemokines, such as IL-3, IL-12, or IL-10 etc., are released or generated in this inflammatory response. Even while AD patients have high levels of Aβ accumulation, they do not suffer from significant cognitive loss when high amounts of IL-12 are also present in the brain, and nearly no tau tangles are also seen in the brain. Additionally, raising levels of IL-12 and IFN-γ may halt or delay cognitive loss, and IL-3 released by brain astrocytes could rewire neuroinflammation by removing extracellular Aβ plaque buildup and preventing the development of intracellular tau tangles (McAlpine et al., 2021; Yang et al., 2022). However, the precise molecular mechanism behind the aforementioned degenerative development is still unknown.

Consequently, neuroinflammation brought on by neurovascular changes, along with immune cell heterogeneity, BBB integrity, the impact of glial cells, etc., play crucial roles in regulating the progression or delaying the onset of neurodegenerative diseases in the brain, which may offer new therapeutical approaches for clinical patients.

## Author contributions

WC, JS, and ZT composed the manuscript. CP helped to revise the manuscript. All authors contributed to the article and approved the submitted version.

## Conflict of interest

The authors declare that the research was conducted in the absence of any commercial or financial relationships that could be construed as a potential conflict of interest.

## Publisher's note

All claims expressed in this article are solely those of the authors and do not necessarily represent those of their affiliated organizations, or those of the publisher, the editors and the reviewers. Any product that may be evaluated in this article, or claim that may be made by its manufacturer, is not guaranteed or endorsed by the publisher.
